# An abductive inferential system approach to weight of evidence syntheses: the problem and military exposures worked example

**DOI:** 10.1080/2833373x.2025.2607260

**Published:** 2026-02-04

**Authors:** J. Scott Parrott, Shobhik Chakraborty, Frederick Coffman, Albert Heuer, Jane Heetderks-Cox, Tami Piemonte, Chelsea Rodriguez, Jacquelyn Mares, Sarah Levan, Salvatore Butera, Marissa J. Coloske, Michael Falvo

**Affiliations:** a School of Health Professions, Rutgers University, New Brunswick, New Jersey, USA; b Us Veterans Administration, Airborne Hazards and Burn Pits Center of Excellence, East Orange, New Jersey, USA; c Truman School of Government and Public Affairs, University of Missouri, College of Health Sciences, Columbia, Missouri, USA; d University of Mississippi Medical Center, University of Mississippi School of Medicine, Jackson, Mississippi, USA; e Milken School of Public Health, The George Washington University, Washington, D.C., USA; f New Jersey Medical School, Rutgers University, New Brunswick, New Jersey, USA

**Keywords:** Airborne hazards, burn pits, evidence synthesis, methodology, military exposures, weight of evidence

## Abstract

**Objective:**

Weight of Evidence methods for evidence synthesis of etiological epidemiology questions regarding historical complex exposures have been developed in recent years but are still in their nascency. This manuscript describes a novel extension of existing methods to address situations where the exposure is historically bound, direct evidence is limited, and new research is unlikely. Principles are illustrated via a worked example in the military exposures context.

**Methods:**

Several existing methodological approaches were adapted and extended using an abductive reasoning approach. We demonstrate an approach to developing a logically interrelated system of PECO questions, integrating and evaluating multiple evidence streams to support inference to the best explanation.

**Results:**

A synthetic advancement on current WOE methods using an “inference to the best explanation” approach provides benefits over more traditional (deductive) methods of evidence synthesis. Concrete military exposure examples are provided.

**Conclusion:**

Applying this approach can provide a principled and transparent method of evidence synthesis to direct policy decisions under conditions of limited evidence and uncertainty.

## Introduction

Upwards of 4.5 million US military personnel were deployed to the Southwest Asia theater of operations (SWATO) between 1990 (Operation Desert Shield/Desert Storm) and 2021 (Operation Iraqi Freedom [2003–2011], Operation Enduring Freedom in Afghanistan [2001–2014], Operation Freedom’s Sentinel [2015–2021]) and related conflicts of this era (Congressional Research Service 2024; Garshick et al. 2019; U.S. Army Center of Military History 2024).

Research on the possible effects of exposure to airborne hazards began shortly after the beginning of the Gulf War in 1990 (Draxler, McQueen, and Stunder 1994) and has continued over the course of the next nearly 35 years, expanding dramatically following the start of the conflicts in Iraq and Afghanistan following the 9/11 attacks. A growing number of veteran health concerns (Sanders et al. 2005) prompted the US government to carry out regular environmental monitoring within the theater of operations. This culminated in a final Department of Defense (DOD) report and two articles by Englebrecht and colleagues documenting general environmental exposures (Engelbrecht et al. 2008; 2009a, 2009b). However, general environmental exposures were only one concern. The major airborne hazard of public concern was exposure to open-air burn pits (Garshick and Blanc 2023). These burn pits were widely used across the theater, with up to 86% of bases documented to have used these as a method of waste disposal, especially between 2005 and 2012 (Woskie et al. 2023). A broad mix of waste, including human excrement, medical waste, food waste, vehicles, computers, lithium-ion batteries, plastic waste, Styrofoam, insecticide canisters, DEET-soaked items, and other potentially harmful substances were disposed of using this method. Using jet fuel as an accelerant, this combustion released a complex mixture of toxins such as volatile organic compounds (VOCs), polycyclic aromatic hydrocarbons (PAHs), heavy metals, furans, sulfur and nitrogen dioxide, hydrogen cyanide and many other toxins at levels often substantially exceeding US Environmental Protection Agency (EPA) levels. Many of these substances are known carcinogens and toxins that have been linked to a spectrum of disorders and pathologies in a variety of tissues (Garshick and Blanc 2023), but they particularly affect the lungs as inhalation is by far the most common mode of entry.

In order for the VA to prioritize disease treatment or provide disability benefits, the disease or condition must be considered “service related” (U.S. Department of Veterans Affairs n.d.). Without sufficient research providing evidence of a “generally causal” relationship (Samet and Bodurow 2008) between exposure and a disease, veteran healthcare and welfare are threatened.

Despite drawing on decades of epidemiological research available to that point on deployed service members and Veterans, the National Academies of Science, Engineering, and Medicine (NASEM) concluded in their 2020 report:

“...there is inadequate or insufficient evidence of an association between airborne hazards exposures in the Southwest Asia theater and the subsequent development of [any of the examined interstitial lung diseases or constrictive bronchiolitis].” (National Academies of Sciences Engineering and Medicine (U.S.). Committee on the Respiratory Health Effects of Airborne Hazards Exposures in the Southwest Asia Theater of Military Operations et al., 2020)

Four years later, having re-examined that body of data along with additional research published since 2020, our team concluded:

Based on research drawn from preclinical, clinical, environmental, and epidemiological evidence streams, *the sum total of the evidence indicates* that deployment to the Southwest Asia theater of operations (SWATO) increases the risk of developing a subset of interstitial lung diseases (ILDs) and constrictive bronchiolitis.

Notice that not only are the conclusions different, but that our conclusion implies a causal relationship (as opposed to the NASEM’s “association”). Why the difference?

Although intervening research played a role, the core differences between the two conclusions stem directly from a difference in evidence synthesis approaches. It was not simply that NASEM failed to utilize any of the existing evidence synthesis approaches designed to provide a basis for making inferences regarding causal relationships between environmental exposures and health conditions, but their synthesis suffered from the critical inadequacy (in this instance) of attempting to rely on the standard hypothetico-deductive method of reasoning typical in evidence synthesis. In other words, their method of inference was inappropriate for the problem at hand.

But why was deductive reasoning (the staple of standard systematic review methods) inadequate in this circumstance? In addition to the typical sparsity of direct human evidence in environmental exposure studies (Whaley et al. 2022), the particular combination of exposures and conditions for airsborne hazard exposure in SWATO is historically bounded. That is, we are limited by exposures that happened under extreme circumstances during a specific span of time in which individual-level exposures were not measured. And, while there are environmental analysis studies of particular locations (see above), they are still limited (in terms of geography and toxicants examined) and cannot be clearly mapped to individual-level exposures (due to troop mobility, variations in duties across time, etc.). In short, direct and reliable measures of exposure are extremely limited, and given the end of the conflicts in 2021, it is unlikely that they will ever be available.

Put somewhat more formally, the typical way of reasoning in evidence synthesis is (as a first step) deductive: if X causes Y in population P, then we can deduce that the data (evidence) should have the following Z pattern. The second step is confirmatory: the evidence does indeed have Z pattern, so the premise (that X causes Y in Z population) is supported. But when no or severely limited data exist, the deductive step is unlikely to provide a useful set of expectations.

What was needed was an approach that was able to account for a large and diverse body of evidence and that, nonetheless, contained very little direct evidence. In short, the approach needed to draw on *abductive*, rather than *deductive* inferential methods to identify a set of expectations that could then be empirically confirmed—we needed a transparent method for reasoning to the best explanation that could lead to a reproducible estimate of confidence in the answer, even in the face of uncertainty. With this approach, assuming the PECO hypothesis is true, we ask what are other “states of affairs” that, if true, would make the PECO hypothesis more likely than an alternative explanation? The key point is that these “states of affairs” are not logically deducible from the PECO hypothesis and so must be inferred on other grounds (which we describe in more detail below). The important difference is not in the confirmatory or ampliative step of evidence synthesis reasoning, but on the way that the set of expectations that guide the definition of “what counts as evidence” are defined.

This presented us with the peculiar situation of having to rely almost exclusively on multiple evidence streams would typically be called “indirect” evidence (Schünemann, Tugwell, et al. 2013; Whaley et al. 2022). While the typical GRADE approach when answering treatment questions differentiates RCT from non-randomized studies (NRS) evidence streams, we found this simple dichotomy insufficient to adequately characterize and evaluate the contribution of different types of evidence to the question regarding environmental exposures. Additionally, while progress has been made toward systematizing the method for integrating information from different evidence streams (Suter et al. 2020; Whaley et al. 2022), none were completely satisfactory for our purpose. For instance, if we take the general GRADE approach of rating up/down based on specified criteria, then “how much” would, say, having strong evidence that the toxicants of interest were present in the theater at the time of deployment, increase our confidence that exposure to airborne hazards in SWATO is causally related to interstitial lung diseases? Or, if we found weak evidence that differential levels of the particulate matter of interest were present in the lungs of deployers versus non-deployers but strong evidence for histopathological differences between deployers and non-deployers, how do we weigh the contribution of both those pieces of evidence (both, using GRADE criteria, “indirect” (Schünemann, Brozek, et al. 2013)) when reaching a conclusion about our primary hypothesis? We found that we needed an approach that allowed us to first weigh the evidence for a range of related questions and then evaluate alternative explanations in light of those findings—a two-step approach to reasoning to the best explanation for what we were seeing.

Our goal was not to try to come up with some alternative evidence synthesis method, but, rather, to build on and extend existing evidence synthesis principles—specifically elements of GRADE methodology (Morgan et al. 2016), the National Toxicology Program’s Office of Health Assessment and Translation (OHAT) method (Office of Health Assessment and Translation 2019), and the EPA’s Integrated Risk Information System (IRIS) (Center for Public Health and Environmental Assessment 2022). Finally, we sought to ground all of this within a “pragmatist” theory of evidence (Reiss 2015) (a pluralistic but systematic approach to inference). This manuscript presents that approach. We seek to provide a justification for the different steps in the method as well as a rationale for departures from the above evidence synthesis approaches when appropriate.

In order to avoid an overly technical and abstract presentation of our method, we use as an example our project examining the relationship between airborne hazard exposure in the SWATO and the development of interstitial lung diseases (ILDs). This manuscript is not a report of our findings for that project. Rather, our focus is on the method with concrete examples provided to ideally make the principles clearer for the reader.

**Figure 1.**
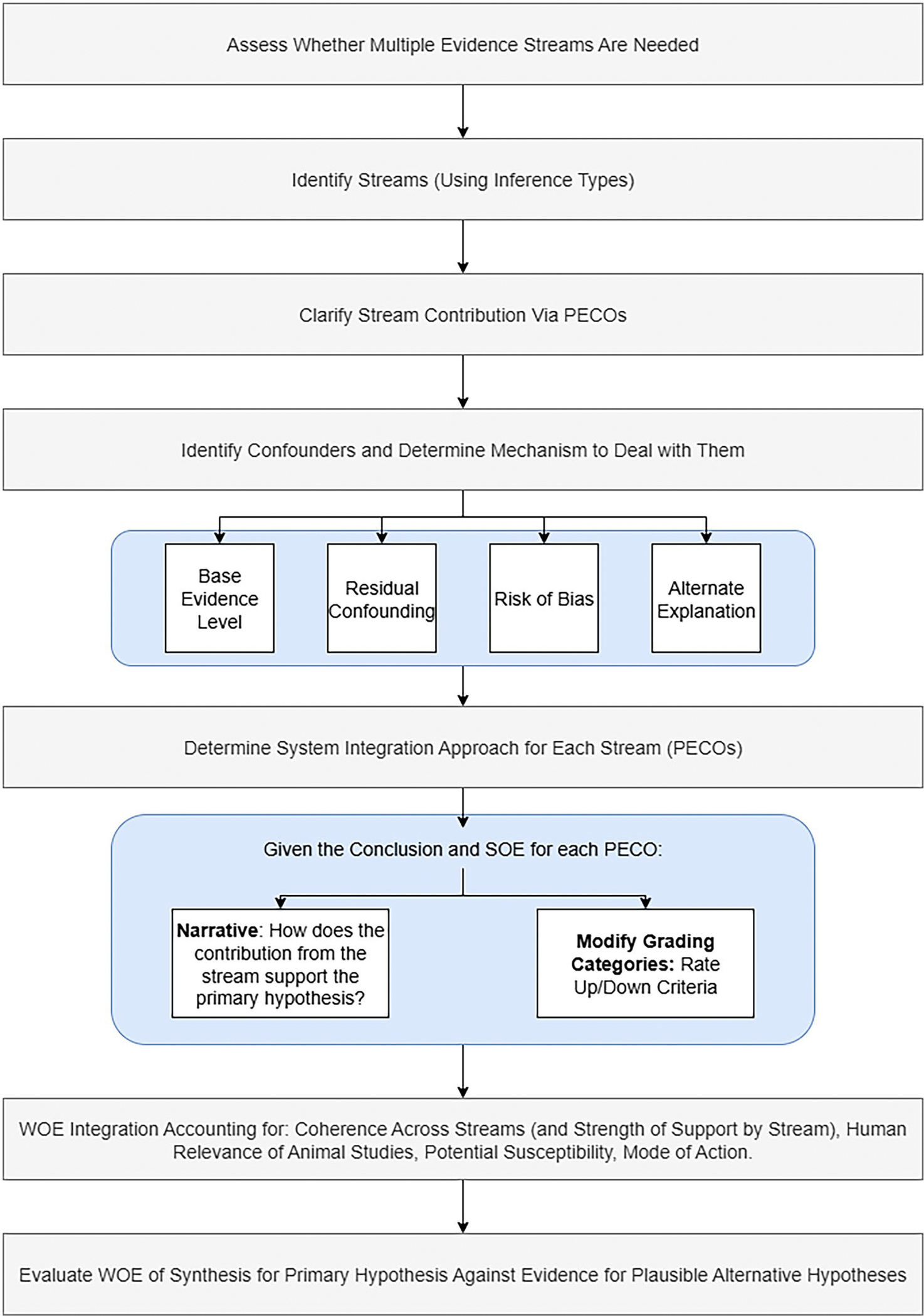
Steps in an inferential system approach to weight of evidence synthesis.

We structured this manuscript to follow the flow of the method we used. See Figure 1.

## 1. Context and approach

Before we describe the specific steps of our method, we need to lay the foundation for our approach. We emphasize that the theory of inference we adopt is more than just gilding on the method. Rather, it shaped our initial approach from the beginning as well as provided the rationale for diverging from some of the more familiar approaches to environmental exposure evidence synthesis.

### 1.1. Theory of inference

We began with the work of Juian Reiss on the logic of inference:

A good theory of evidence should explicate both **support** and **warrant**. We need, on the one hand, criteria or guidelines that tell us **what kinds of facts** we have to collect in order to evaluate a hypothesis; we need to know what facts are relevant to the hypothesis. We need, on the other hand, criteria or guidelines that tell us how to assess the hypothesis, given the facts we’ve collected in its support, or, conversely, criteria or guidelines that tell **us how much support of what kind we need in order to achieve a given degree of warrant.** (Reiss 2015) [emphasis ours]

So, first, we needed to identify what kinds of facts (evidence) were relevant, and second, we needed an approach that could operationalize how and “how much” support each type of evidence might provide to reach a specified level of confidence (degree of warrant).

Both of these questions are answered with reference to the primary question or hypothesis. For our example:

#### Primary Question:

What is the evidence to indicate that exposure to the range of airborne hazards present in the SWATO is causally related to the development of interstitial lung diseases?

Or, expressed as a hypothesis:

#### Primary Hypothesis:

Exposure to airborne hazards during deployment to SWATO increased the risk of the development of at least some interstitial lung diseases.

We recognize that some readers may be surprised by our focus on causality rather than (as is more typical with an epidemiological question) “association.” Our focus on causality is intentional and follows the 2008 NASEM charge (Samet and Bodurow 2008) for Veterans Administration decision-making regarding presumptive injury:

In making a decision to provide compensation, VA needs to determine whether the illness of concern can generally be **caused** by exposures received during service and whether the illness in a specific claimant was caused by the exposure. The answer to the general question of causality comes from a careful review of all available scientific information.… (7) [emphasis ours]

We note that this is not necessarily *proof of* causality, but rather an inference regarding the plausibility of causality given the evidence. In NASEM’s words,

If an association has been established, the next (and more difficult) task is to assess the evidence for causation by trying to eliminate alternative explanations to causality for the association. (160)

So, unlike other evidence synthesis efforts that seek proof of causality (as is typical with treatment questions via RCT using deductive reasoning methods), our charge was to determine whether a causal relationship was generally plausible. So, what would constitute a warranted inference of causality?

#### 1.1.1. Inferential system

As Reiss points out in his inferentialist account of causation (Reiss 2012), the question of “How can we know that a proposition is true?” is integrally linked to the proposition’s meaning. And, in turn, the meaning of a proposition is given by its *relationships to other propositions*—what it follows from and what follows from it within the boundaries of a community of discourse. In other words, the truth of a proposition resides not in the proposition itself, but in the ***system*** of propositions (statements) in which it is embedded.

Take our primary hypothesis: “Exposure to airborne hazards in the SWATO is causally related to the development of interstitial lung disease”. We evaluate the plausibility of this assertion relative to the plausibility of a series of other propositions that are logically related to it. For example:

- **Antecedence**: The plausibility of our primary hypothesis depends on the plausibility of a related proposition: A range of airborne toxicants is present in the SWATO. If this statement is found to be implausible, then the truth of the primary hypothesis is untenable.
- **Specific Differences**: If the primary hypothesis is plausible, then we would expect that the following proposition is plausible in light of the available evidence: “The incidence of ILDs in military personnel deployed to SWATO is higher following deployment than before” (temporal). We would also expect the following to be plausible: “Lung infiltrates known to be related to toxicants present in SWATO are present in the lungs of deployers in greater concentrations than in the lungs of similar non-deployed military personnel” (proximal). Similarly, “There is a higher prevalence of ILDs in deployed versus non-deployed military” (differential outcome) would likewise need to be shown to be plausible (supported by available evidence).
- **Non-locality**: While a causal relationship can hold within a specific context, evidence that the proposed causal relationship is robust across contexts provides additional support. Thus, “In civilian populations, individuals with higher exposure to airborne toxicants have higher rates of ILDs than individuals with lower exposure” (analogy) likewise provides support for the primary hypothesis.

The above are but a few examples of the propositions that, should they be plausible, may increase (or decrease, depending on the evidence) the warrant for asserting the truth of the primary hypothesis. Again, none of the above (or other) related propositions *ensure* the truth of the primary hypothesis, but they do increase its plausibility, or, better yet, our confidence in the truth of the primary hypothesis, especially when evaluated against alternative plausible hypotheses (explanations). These related (or entailed) hypotheses constitute a system that reflects back on the primary hypothesis, strengthening or weakening our confidence in the plausibility of the primary hypothesis.

The first challenge, from an evidence synthesis perspective, is then to define (a priori) or specify the set of propositions that constitute the abductive inferential system of the primary hypothesis.

## 2. Evidence streams: identifying and clarifying

**Figure 2.**
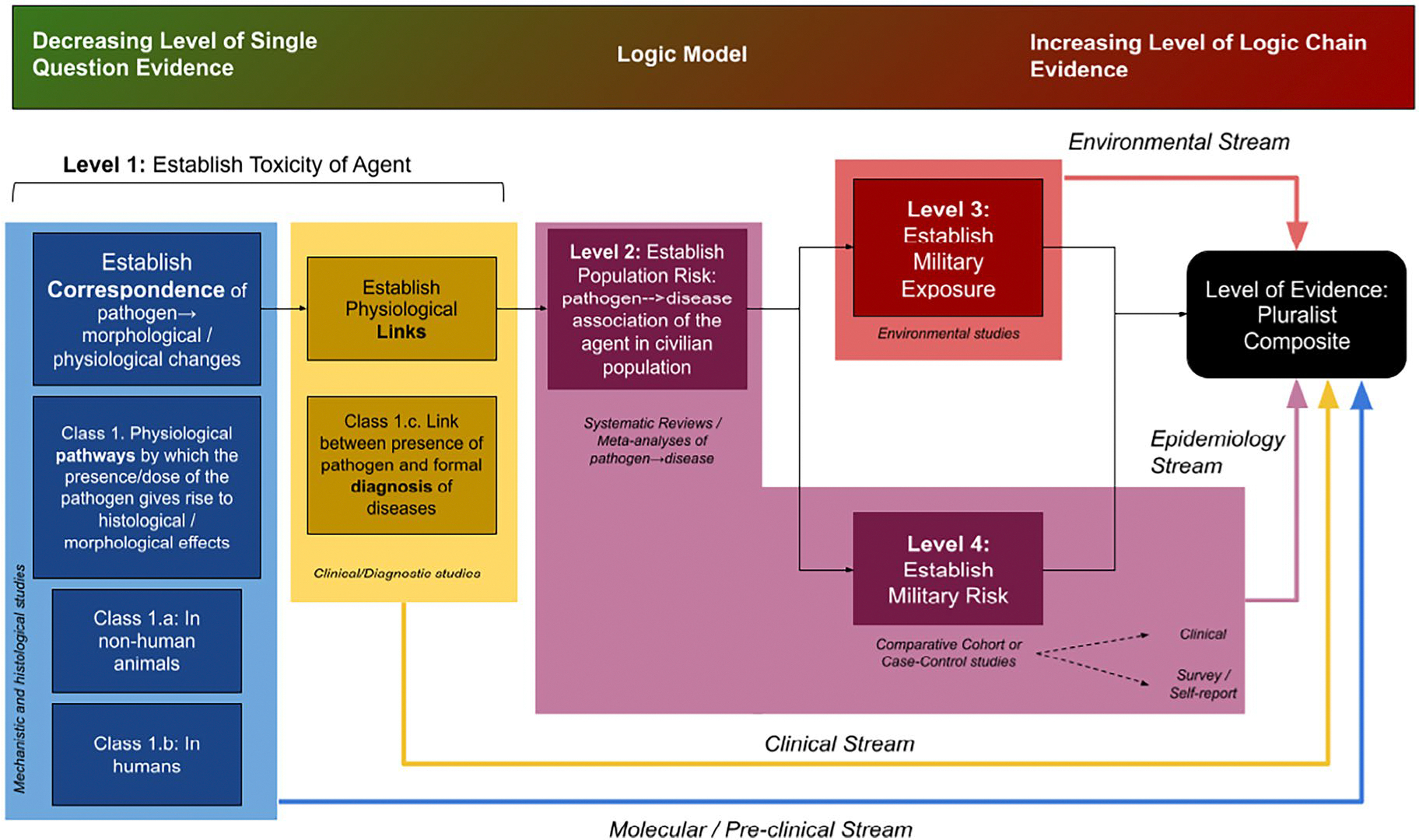
Evidence streams used to evaluate military *risk.*

The need for additional evidence streams was made clear by the 2020 NASEM report (which focused almost exclusively on epidemiological research) (National Academies of Sciences Engineering and Medicine (U.S.). Committee on the Respiratory Health Effects of Airborne Hazards Exposures in the Southwest Asia Theater of Military Operations et al. 2020). Additionally, this report, as well as the 2008 NASEM presumptive injury guidance (Samet and Bodurow 2008) also recommended integrating preclinical (including animal and in vitro) research. We were also aware of relevant environmental and clinical studies. So, rather than define evidence streams based simply on study design, we focused on different sources of information based on their scope of inquiry: molecular/pre-clinical, clinical, environmental and epidemiological, and then organized them into a conceptual framework that integrated the different streams into a logic chain (Figure 2). This is consistent with the GRADE approach of drawing, when needed or useful, on non-randomized studies (NRS) to provide additional evidence when RCTs are sparse or unavailable. (Guyatt, Iorio et al. 2025; Schünemann, Tugwell, et al. 2013)

This allowed us to (in Reiss’ terms) identify “what kind of facts” were germane. Further, mapping out the relationships between the streams allowed us to define the logic by which “facts” (evidence) from one evidence stream related to the others and to the primary hypothesis.

This was only the first step. We next needed an approach that would clarify the types of support (or disconfirmation) each stream could provide and how this would either strengthen or weaken our confidence in the plausibility of the primary hypothesis. This meant formulating a series of questions (hence, propositions) within each evidence stream. But how to define the interrelated set of propositions that would provide inferential warrant for the primary hypothesis, or, in other words, what questions to ask?

**Figure 3.**
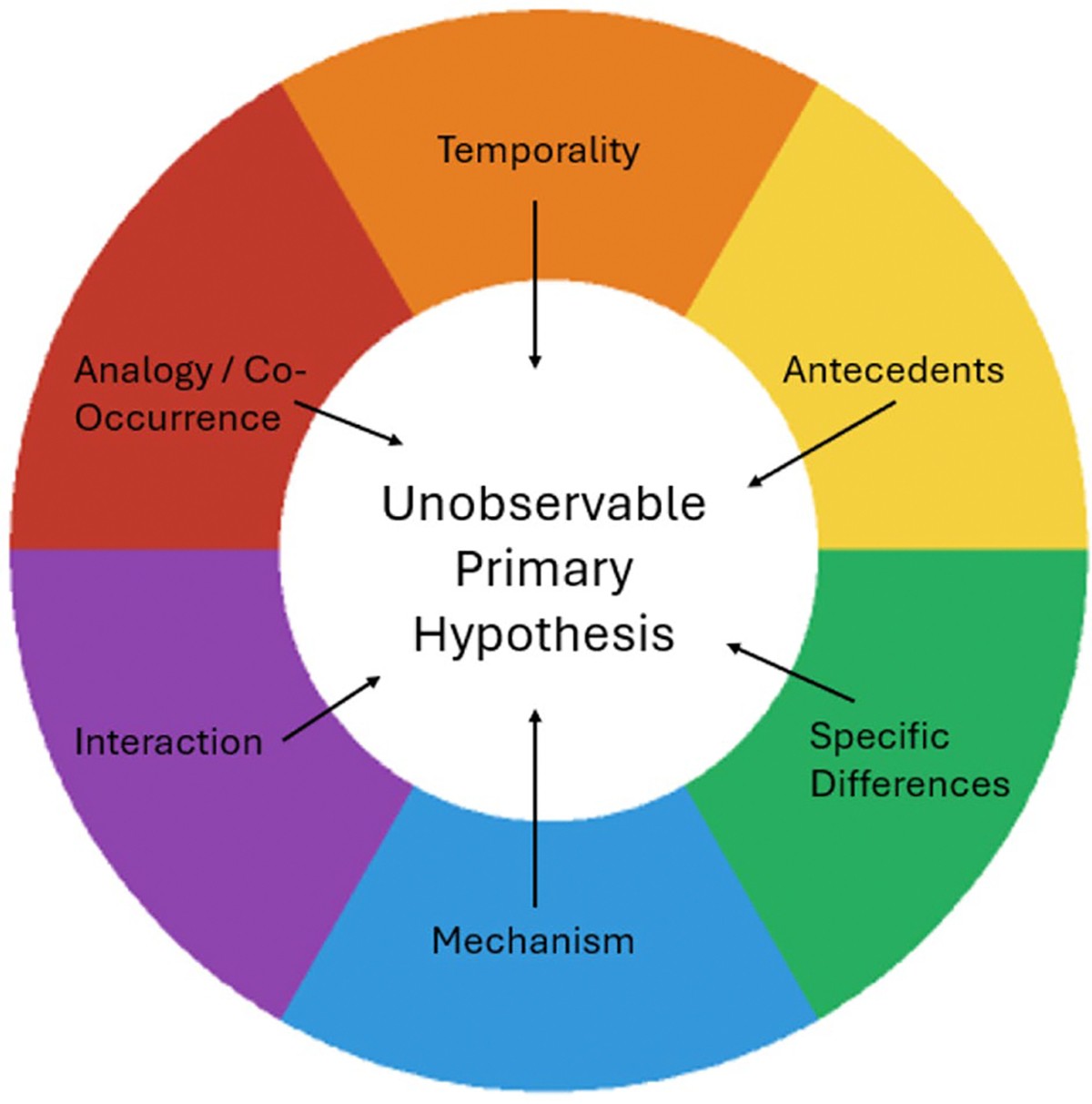
Types of warranted expectations supporting inferences regarding the primary hypothesis.

While we doubt that there is a single best way to define the system of related propositions, we took our cue from others who have used Bradford-Hill’s criteria for causation as the basis for evaluating or supporting causal plausibility in an evidence synthesis context (Howick, Glasziou, and Aronson 2009; Schünemann et al. 2011; Suter et al. 2020; Vincent et al. 2024). This provided a set of inferential categories that defined a system of “warranted expectations” that should be true (i.e., verifiable) if the primary hypothesis were plausible (Figure 3).

These categories provided a conceptual framework that helped us identify *how* evidence within each of these categories would contribute to our confidence regarding the primary hypothesis, as well as provided the basis for formulating specific PECO-type questions within each type (Table 1).

Notice that none of the above categories of evidence (or the key questions) specifically address the primary hypothesis, but are all implicated within the inferential space (the system of propositions) of the primary hypothesis. This, then, constituted the inferential system that guided our work.

**Figure 4.**
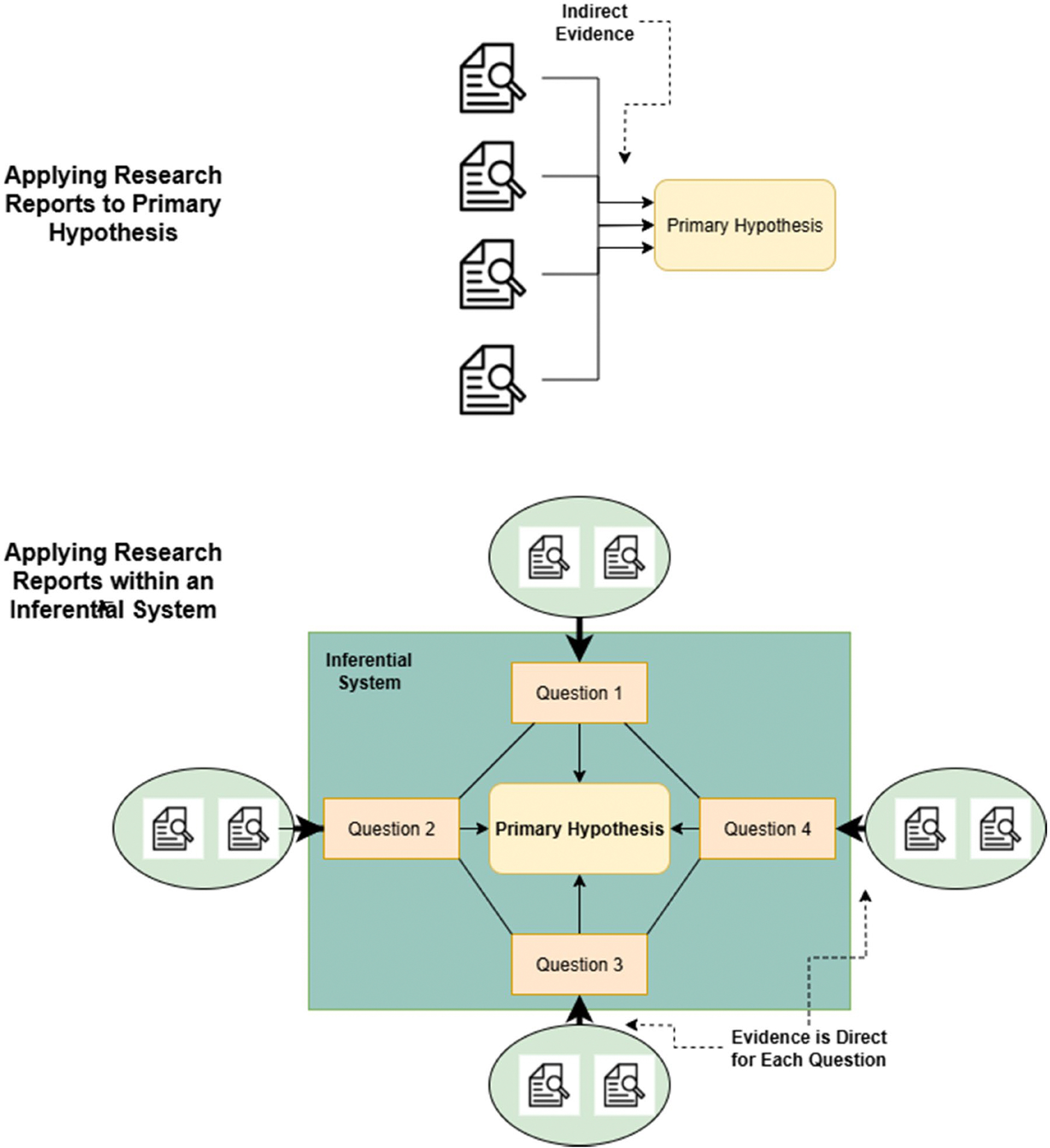
Indirect versus direct evidence in an abductive inferential system approach.

The independence of these inferentially related key questions is an important point. In the GRADE method, the judgement regarding the directness or indirectness of evidence is relative to the PICO/PECO question (Guyatt, Iorio et al. 2025; Schünemann, Tugwell et al. 2013). Thus, while evidence from a key question in one inferential category would be considered “indirect” if used to answer a question in another inferential category (or if applied directly to the primary hypothesis), by splitting out a series of separate PECO questions, the evidence used to answer that question can be limited to direct evidence. This means that the individual research reports were *not* used to attempt to directly draw conclusions regarding the plausibility of the primary hypothesis. Rather, the individual research articles are, as it were, a “step removed” within the abductive inferential system approach. It is the relationship between the key questions and the primary hypothesis (rather than the relationship between individual research reports and the primary hypothesis) that serves to strengthen or weaken our confidence in the plausibility of the primary hypothesis. In other words, the combination of the system of key questions (and the strength of evidence for each) provides the warrant for the primary hypothesis. For example, while the research reports regarding differences in lung pathology would be indirect relative to the primary hypothesis, they would be direct with respect to the specific question within that inferential category (Figure 4). This distinction will prove particularly important in the evidence integration at the weight of evidence stage.

## 3. Mechanisms for integrating information from confounders

Confounding is a serious issue in the synthesis of observational studies, and in our situation, this problem was particularly challenging due to the multiple known confounders. While some were known to lead to bias toward the null, the effect of other confounders was less clear. Additionally, not all confounders were relevant for all questions. The three most pervasive confounders identified in the deployed military exposures are listed in Table 2.

The challenge we faced was how to evaluate the cross-cutting effects of these different confounders when evaluating the series of inferential system questions, as well as the implications when combining the findings into the larger weight of evidence assessment for the primary hypothesis. For instance, given that both effect dilution and the healthy warrior effect were relevant for many of the questions, what would this mean for rating up (Guyatt, Wang, et al. 2025) when evaluating the strength of the evidence?

On our reading of the above approaches for handling confounders in environmental exposures evidence synthesis, there are three possible ways that information from confounders can serve to modify our confidence in the evidence. We explain the basis for each and how each figured into our evaluation of the weight of evidence.

1. **Residual Confounding Toward the Null:** This is a well-established principle for the GRADE approach. When an uncontrolled confounder is expected to bias toward the null, then evidence of an effect is given greater confidence (Brozek et al. 2021).
2. **Risk of Bias:** Increased risk if a significant proportion of studies do not adequately adjust for a confounder (Guyatt, Wang, et al. 2025).
3. **Base Evidence Level:** Recent GRADE guidance suggests the possibility of starting with a higher than “low certainty” when the risk assessment tool explicitly takes into consideration selection bias and confounding with reference to an idealized reference RCT (Schunemann et al. 2019) (see the endnote for a description of an imagined reference RCT).

### 3.1. INUS causality and the importance of evaluating alternative explanations

In addition to the above, we followed Reiss’ principle (Reiss 2015) that data incompatible with an alternative hypothesis provides indirect support for the primary hypothesis. Thus, in addition to the above principles for “adjusting confidence” in the evidence (and following the 2008 NASEM guidance regarding alternative explanations (Samet and Bodurow 2008)), we specified what we would expect to see under the most plausible alternative hypothesis (see below) and when the evidence was incompatible, this served to strengthen our inference that airborne hazard exposure during deployment was the most plausible explanation. This is consistent with the “inference to the best explanation approach to epidemiological reasoning (Krieger and Davey Smith 2016; Lipton 2017).

Evaluating an alternative explanation for each PECO question (as appropriate) within the inferential system may seem like an inordinate amount of work. To this, we have two responses. First, in contexts where the purported cause (exposure to airborne hazards) is an insufficient but necessary part of a condition that is itself unnecessary but sufficient for the result (INUS causality (Palenberg 2023; Whitaker et al. 2020)), it is vital to rule out any alternative explanation that (a) is itself purported to be necessary for the outcome and (b) is not entailed by the exposure (cause) of interest. (For a formal proof of this necessity and its value for making inferences to guide policy, see Supplemental Material). Second, the practical evaluation of this principle is much less onerous than it might superficially appear (as we demonstrate below). Indeed, we argue that one of the key flaws of the NASEM’s approach to evaluating the research regarding exposure to airborne hazards and interstitial lung diseases (in addition to failing to adequately account for multiple evidence streams in a systematic way) was precisely because they did not evaluate any alternative explanation. Reasoning to the best explanation could have allowed them to conclude that even though the evidence they had available for deployment-related airborne hazard exposure may have been relatively weak, it was still the most plausible explanation for the higher rates of ILDs among deployed personnel. This abductive approach could have resulted in a more intuitively plausible conclusion.

### 3.2. Different approaches to adjusting for known biases and confounders

Using the above principles, we then made the following adjustments at the different levels of judgement (article level [ROB], question level [strength of evidence (SOE)], and system level [weight of evidence (WOE)]):

- **Exposure Misclassification (Effect Dilution), Baseline SOE Level:** Because this confounder was ubiquitous, we reasoned that findings indicating higher risk in deployers detected by the observational research were likely biased toward the null. When germane to the question, a baseline level strength of evidence (SOE) rating of “moderate” (using the IRIS levels of certainty (Center for Public Health and Environmental Assessment 2022)) was used if the evidence base for that question consisted of higher confidence observational designs (e.g., cohort or case control versus cross-sectional or case series). This decision is consistent with the principle proposed in Schunemann et al. (2019) when the selection bias and confounding are integral to risk of bias assessment in NRS studies.
- **Comparator Selection (Healthy Warrior Bias), Adjusted SOE Level (Residual confounding):** When appropriate to the question, the presence of healthy warrior bias warranted increasing SOE confidence (via rating up for residual confounding) due to uncontrolled residual confounding.
- **Tobacco Use, Adjusted SOE Level (Risk of bias):** Certainty adjustment could be modified in two ways with respect to the effects of smoking (the direction and magnitude of which were uncertain). First, studies that did not adjust for smoking were automatically rated as “probably” or “definitely” at high risk of bias (following the OHAT judgements for signaling questions) (Office of Health Assessment and Translation 2019). If a majority of studies relevant to the question were not adjusted for tobacco use, this would result in a downrating at the SOE judgement level.
- **Tobacco Use as Sole Explanation, WOE Level:** For each question, we explicitly evaluated whether smoking during deployment was sufficient to explain the observed patterns. The reasoning was that if smoking, of itself, could explain the findings, then whatever support that question might provide for the primary hypothesis was diminished. However, if the findings were incompatible with smoking as the sole cause (i.e. ruling out the effect of airborne hazards), then confidence in the primary hypothesis could be increased.

The above principles guided our evaluation of the evidence for each question and that question’s contribution to the overall weight of evidence. However, these principles were applied somewhat differently based on the nature of the question. So, for instance, for questions where deployed versus non-deployed military personnel were not the focus of the comparison (e.g., civilian literature, environmental analyses), the above biases (and thus adjustments) were not necessarily relevant.

## 4. Question strength of evidence and question integration

Different searches were used to identify evidence from the different evidence streams, and then typical systematic review methods were used to design the extraction templates, extract, and synthesize the data.

We used the GRADE approach of first establishing a baseline level of confidence for the evidence to each question within the inferential system (though, as noted above, we did not use the simple RCT=High and NRS = Low approach, but, rather, adjusted the baseline level based on the appropriateness of design and confounder handling (Schunemann et al. 2019)) and then rating up or down in confidence based on specified GRADE criteria—with a single exception: mechanistic research.

### 4.1. Mechanistic research: a special case

Recent GRADE advancements provide more nuanced perspectives regarding when mechanistic research (biological plausibility) might be of more or less concern regarding indirectness. However, mechanistic research is still conceived in terms of *surrogacy* (where the mechanism or biomarker is used as a proxy for the outcome of interest) (Morgan et al. 2016; Whaley et al. 2022). Within the context of a typical approach where mechanistic research would be applied directly to the primary hypothesis, rather than, as we suggest, to a related PECO question within the inferential system, this makes some sense.

The question then becomes a matter of determining how the findings from mechanistic PECO questions should be integrated with other questions within the abductive inferential system. This amounts to asking: how do the mechanistic findings influence our confidence with respect to the findings from other evidence streams? A case in point: we may find suggestive though limited evidence for histopathological differences (specifically regarding increased fibrotic activity in the lung and dysfunctional adaptive immune responses). How would mechanistic research demonstrating differential mRNA changes associated with adaptive immune responses leading to increased lung fibrosis in deployers versus non-deployers influence our confidence in the histopathological results? Following pragmatist inferential principles, this would give us warrant to expect that if we see differential histopathological patterns, then we *should expect* to find evidence that maladaptive immune responses give rise to these differences. This means that:

- Were we to find no evidence of maladaptive immune responses, this would neither strengthen nor weaken our inference about the difference in histopathology (the research may simply not be available).
- In contrast, if there is mechanistic evidence that innate and adaptive immune responses lead precisely to the observed histopathological differences, this would **increase** our confidence in the histopathological evidence.
- Similarly, if we found mechanistic evidence suggesting that adaptive immune responses were unlikely to lead to the observed histopathological differences, this would **decrease** our confidence.

Thus, the results of the mechanistic research have a direct bearing on our confidence (strength of evidence) of questions regarding the relationship between a “root cause” (an exposure or intervention) and a target outcome (typically a diagnosis or condition, also called the apical outcome). Because mechanistic research specifies the intermediate causal physiological chain of events linking the root cause to the target outcome, it has a *direct* bearing on our confidence.

In light of this, for some questions (where evidence from alternative evidence streams may specify the intermediate causal chain), we added an additional *coherence* “rate up/rate down” criterion to the adapted GERADE strength of evidence criteria. This, we found, was more inferentially plausible and flexible (increasing, decreasing, or no effect on confidence) than simply deciding whether or not to “rate down”.

Having thus created a flexible approach for evaluating the evidence for each of the inferential system questions, the evidence for each question was then synthesized (narratively or meta-analytically, depending on the evidence), and the strength of evidence for that question was evaluated. Additionally, as noted above, we explicitly evaluated the evidence for the most plausible alternative explanation (i.e., whether the known increase in smoking associated with deployment could fully explain the observed differences in the research). If a situation was encountered where smoking could not be ruled out as the sole explanation, then our confidence in the findings were decreased. For more detail regarding the importance of evaluating the most plausible alternative, see Supplemental Material.

The next step was to integrate the evidence across questions in the inferential system and draw a conclusion regarding the primary hypothesis.

## 5. Within- and across-stream synthesis and overall weight of evidence

Precisely, how was evidence from the different streams integrated? The OHAT (Office of Health Assessment and Translation 2019) and IRIS (Center for Public Health and Environmental Assessment 2022) methods differed somewhat in their approach, and we determined that a hybrid of the two methods would best serve our purpose.

Following OHAT, at the strength of evidence (SOE) level:

- **Baseline confidence.** We used study characteristics to establish a baseline level of confidence. Only bodies of evidence that met all four of the following characteristics could be assigned a baseline level of “high” confidence: controlled exposures, exposure prior to the outcome, individual outcome data, and comparison group used. For the military exposures, all but the first criterion were possible, and so the baseline level of confidence was generally “moderate” (based on the effect dilution confounding [see above] and consistent with the recommendations of more recent GRADE guidance when risk of bias directly addresses confounding (Schunemann et al. 2019)).^1^
- **Coherence domain.** We included Coherence as a “rate up” domain in the Strength of Evidence (SOE) assessment when appropriate. This allowed us to take into account cross-species consistency, biological (mechanistic) plausibility, and cross-population consistency when estimating a level of confidence. If the consistency or biological plausibility criteria were met, the expert panel could decide to rate confidence up a level.

Adapting the IRIS and Suter et al. methods at the WOE level:

- **Compared evidence for alternative explanations across key questions.** Across the key questions, we evaluated which (if either) of the alternative explanations did the best job of accounting for the available evidence (i.e. exposure to environmental airborne hazards versus smoking).
- **Number, diversity, and coherence.** Drawing from Suter et al. (2020), the number and diversity of studies, as well as the consistency or coherence between the findings for each key question, provided a basis for rating up or down confidence in the overall conclusion.
- **Mechanistic support.** Human relevance of findings in animals, potential susceptibility, and understanding of biological plausibility and mode of action were assessed (Center for Public Health and Environmental Assessment 2022). This provided a basis for increasing or decreasing confidence in the overall conclusion.
- **Direction of known biases.** A range of known biases (e.g. healthy warrior bias, exposure misclassification (dilution bias), outcome misclassification, etc.) was specified a priori, and the direction of the bias was determined based on existing research. Taking into account these biases (especially if there was an empirically identifiable bias toward the null) provided a basis for increasing or decreasing confidence in the overall conclusion.

Thus, by developing a logically integrated system of related PECO questions (where direct evidence for each could be assessed) and evaluating each against the most plausible alternative explanation, we were able to systematically integrate evidence across evidence streams to reach the most plausible and actionable conclusion.

## 6. Discussion

Our investigation of the relationship between past military airborne hazard exposure and lung disease uncovered a host of evidence synthesis challenges but also provided the impetus to develop a principled and novel method for addressing the primary concern described in this paper, viz., is exposure to airborne hazards (burn pits and others) a causally plausible explanation for unusually high rates of ILD in formerly deployed personnel (Jeganathan and Sathananthan 2024)? Approaches that did not evaluate the evidence as a system of logically related PECO questions or concomitantly evaluate alternative conclusions were simply inadequate for syntheses of this nature and complexity. While the methods for WOE approaches are still developing, enough progress has been made in this area that we could adapt and extend several extant approaches to more thoroughly and successfully draw actionable conclusions regarding the relationship between military airborne hazard exposure and interstitial lung diseases. We believe that the approach described in this paper, which integrates multiple evidence streams, specifies key questions based on complimentary inferential criteria, and uses an abductive “inference to the best explanation approach” (see (Lipton 2017)), provides a workable approach for evidence syntheses of environmental etiology, especially when the exposure of interest is historical.

We framed our approach in contrast to that taken by the NASEM panel in their 2020 report (National Academies of Sciences Engineering and Medicine (U.S.) Committee on the Respiratory Health Effects of Airborne Hazards Exposures in the Southwest Asia Theater of Military Operations et al. 2020). We should point out, however, that this is more of a rhetorical contrast than a critique of the work of the NASEM panel. Within the boundaries of their charge (which focused almost exclusively on the epidemiological evidence stream), the work of the NASEM panel was comprehensive and identified a number of limitations in previous efforts to evaluate the connection between airborne hazards (and burn pits specifically) as well as provided concrete suggestions for enhancing the knowledge base (including the recommendation to expand pre-clinical stream research). Indeed, our work was informed by both these recommendations and important research published in the intervening years. The fundamental difference between our approach and that of the NASEM 2020 report, however, that our approach (defining an inferential system and reasoning to the best explanation) does not demand a level of certainty that would truly be feasible only with clinical trials. As we have argued, while the best explanation among the plausible alternatives cannot guarantee certainty, it does provide a principled and transparent basis for drawing actionable conclusions in the face of uncertainty.

Within the context of our research, this alternative approach to synthesis provided a rigorous method for addressing a pressing public health question: is exposure to airborne hazards present in the Southwest Asia Theater of Operations a causal contributor to the unusually high rates of interstitial lung diseases among formerly deployed personnel? By drawing on multiple evidence streams, formulating a logically integrated series of questions that provided multiple bases for inference, and critically evaluating the evidence for alternative explanations, we were able to reason to the best explanation. While there are still many unknowns, we now have a plausible explanation to inform policy and avoid the paralysis of misplaced conclusions of “insufficient evidence.”

The evidence indicates higher rates of respiratory complaints (a point recognized in the 2020 National Academies report (National Academies of Sciences Engineering and Medicine (U.S.) Committee on the Respiratory Health Effects of Airborne Hazards Exposures in the Southwest Asia Theater of Military Operations et al. 2020)) as well as higher rates of interstitial lung diseases in formerly deployed military personnel compared to non-deployers. This begs for an explanation precisely because if exposure to airborne hazards during deployment to the Southwest Asia theater of operation is a contributing cause of the unusually high rates of these diseases in younger individuals, then the evidence can inform the US Veterans Administration policy and clinical decision-making. Using an evidence synthesis approach more suited to complex etiological questions of environmental exposure, which seeks to identify the explanation that best accounts for the larger body of evidence, provides an informed way forward.

### 6.1. Limitations and applications

We see two key limitations to the approach we have described above: boundaries on the level of certainty attainable, and the exploratory nature of this approach.

We hasten to note that we do not propose the approach that we have outlined above as a general purpose alternative to standard deductive evidence synthesis methods for environmental exposures. Deductive methods, which are able to apply strong comparative evidence directly relevant to the question, will always be preferred to abductive methods that rely on different streams of indirect evidence to draw warranted inferences. However, when the situation is such that direct evidence is absent or extremely limited and, because of the historically bounded nature of the exposure, new evidence is unlikely (and where clinical or policy decisions are required regardless of the state of the evidence), our abductive inferential systems approach offers a transparent and principled alternative. Within the policy realm, we liken this to empiric therapy in the clinical realm (i.e., where the starting therapy is based on the most likely diagnosis or pathogen before definitive diagnostic confirmation is available, often using clinical judgment, epidemiology, and risk factors to guide initial choice).

Is certainty necessary? We cannot make a blanket statement about the types of evidence that may be deemed necessary by policymakers in all given areas. Within the context of VA decision-making regarding presumptive injury, our charge was to evaluate whether, with some level of confidence, a causal relationship was plausible and was stronger than the alternative explanations. Other contexts may require different levels of confidence. However, uncertainty will remain. What our approach does add is the ability to transparently assess different levels of confidence using reproducible methods. Using an abductive inferential systems approach, certainty will be relative, not absolute. When this is acceptable, the approach we have defined may be useful.

Second, we offer our approach as an exploratory alternative. That is, while we have attempted to make the steps of our process and our reasoning transparent, we cannot say how an abductive inferential systems approach might be operationalized within other contexts. Refinement and extension are surely necessary. This is but a first step to open up transparent evidence synthesis in situations of extremely limited direct evidence where the answer to the question is, nonetheless, of great public interest. While decisions at different steps in the process will unavoidably be subjective, our hope is that by specifying the steps in the process, the decisions can be made intersubjectively plausible within a community of discourse. Are there better approaches to identifying the related (entailed) hypotheses (PECO questions) than using the Bradford Hill criteria? Perhaps. Are there different approaches for identifying confounders that may serve as alternative explanations when the primary hypothesis has INUS causal status? Given Reiss’ notion that epistemic warrant is relative to the community of discourse and practice working in an area of inquiry, we assume that there must be.

Where and how an abductive inferential systems approach may be applied in other areas of evidence synthesis is for the policy-oriented evidence synthesis community to decide. Our goal has been to offer this approach as a first step in this direction.

## Supplementary Material

Suppl Material

Supplemental data for this article can be accessed online at https://doi.org/10.1080/2833373X.2025.2607260.

## Figures and Tables

**Table 1.** Inferential categories, relation to primary hypothesis, and key questions.

Inferential category	Relation to primary hypothesis (H’)	Question
**Antecedents**: What must logically be true for the primary causal relationship to hold?	Confidence in the plausibility of H’ is weakened if there is no evidence of airborne hazards in the relevant geographical area and strengthened if known toxins are demonstrated to be present.	**Q1:** What evidence exists to provide information on the constituents of airborne hazards in the Southwest Asia Theater of Operations (SWATO)? **Q2:** What evidence exists that airborne hazards present in the SWATO are toxic in humans?
**Temporality:** Is there evidence that the cause precedes the effect in time?	Confidence in the plausibility of H’ is weakened if there is no evidence that the cause precedes the effect in time and strengthened if such evidence is present.	**Q3:** Is there evidence of decreased respiratory health in military personnel following deployment to the SWATO compared to pre-deployment?
**Analogy/Co-Occurrence:** Associations between similar or analogous exposures and outcomes in different populations indicate a similar causal mechanism (e.g., civilian research on occupational and environmental exposures)	Confidence in the plausibility of H’ is weakened if there is no evidence for similar effects in other populations and locations, and strengthened if such research is present.	**Q4:** In the research on civilians, what is the evidence for an association between exposures to airborne hazards and interstitial lung diseases (ILDs)?
**Interaction:** Is there evidence of a direct (physical) interaction between the cause and the effect?	Confidence in the plausibility of H’ is increased if pathogens are confirmed to be present in the lungs of deployers and weakened if pathogens are found to not be present	**Q5:** Are known pathogens confirmed to be present in greater quantities in the lungs of SWATO deployers with the condition of interest compared to non-deployers?
**Mechanism:** Is there evidence that the exposure, if present, has a biologically plausible connection to the outcome of interest?	Confidence in the plausibility of H’ is increased if, across species, biological mechanisms that link exposure to the target set of physiological changes are identified and weakened if such evidence is not present.	**Q6:** In *in vitro* and *in vivo* research, what is the evidence for mechanistic linkages between military-relevant exposure profiles of personnel deployed to the SWATO and inflammatory and fibrotic processes in the lungs? **Q7:** In human biomarker research, what is the evidence that airborne exposures during military deployment to the SWATO result in measurable biomarker differences between deployed versus non-deployed military personnel? **Q8:** In human biomarker research, what is the evidence that airborne exposures during military deployment to the SWATO result in changes in biomarkers specifically implicated in pro-inflammatory and pro-fibrotic pathways (e.g., change in expression of cytokines or miRNAs associated with disease risk)?
**Specific differences:** What empirical differences would we expect to see if the primary hypothesis were true?	Confidence in the plausibility of H’ is strengthened if specific differences (symptoms, pathology, diagnoses) are present to a greater degree in deployers compared to non-deployers.	
	**Differences in symptoms**	**Q9:** Are military personnel deployed to the SWATO more likely to complain of respiratory problems following deployment compared to those who did not deploy to the SWATO?
	**Differences in lung pathology**	**Q10:** Are the lungs of military post-deployers to the SWATO seeking treatment post-deployment for respiratory complaints histopathologically distinct from: **Q10(a):** Healthy Individuals? **Q10(b):** Military personnel who did not deploy to the SWATO? **Q10(c):** Individuals with other respiratory diseases?
	**Differences in diagnoses**	**Q11:** Are military personnel deployed to the SWATO more likely to receive an ILD diagnosis following deployment than those who did not deploy to the SWATO?

Note: SWATO: the Southwest Asian Theater of Operations.

**Table 2.** Key confounders for military exposures questions.

Confounder	Description	Direction of effect	Magnitude
**Exposure Misclassification** (Effect Dilution)	Treating all deployers as a homogenous exposure group likely underestimates the effect in military personnel most directly and consistently exposed.	Bias toward the null.	Based on research differentiating military personnel by combat roles and occupations (Rivera et al. 2018), this underestimate could be as much as 26%–54%.
**Comparator Selection** (Healthy Warrior Bias)	The US Department of Defense policy stipulated that only military personnel without respiratory issues were eligible to deploy. Additionally, physical training standards had to be met for deployment. This resulted in healthier military personnel in the exposure group (Haley 1998).	Bias toward the null.	This bias could result in an underestimate of as little as 65% to as much as 102% (Barth et al. 2014).
**Smoking** (Co-Exposure)	Smoking is known to cause histopathological, immune, and genetic changes associated with lung disease.(Obernolte et al. 2022) Smoking has also been shown to be slightly higher in deployed versus non-deployed US military. (Harte, Proctor, and Vasterling 2014).	Uncertain direction of effect of bias.	Civilian literature finds that not adjusting for smoking may result in an underestimate of various ILD conditions by 10–40% depending on the type of exposure (Pauchet et al. 2022). In contrast, evidence in the military literature examined for this project (meta-analysis of asthma) suggests that not adjusting for smoking may result in an overestimate of approximately 23%.

## Data Availability

All extracted data was formerly available at US Agency for Healthcare Research and Quality (AHRQ), Systematic Review Data Repository Plus (SRDR+): https://srdrplus.ahrq.gov/. Note that in November 2025, due to a change in US policy, SRDR+ was decommissioned. As of the time of writing, efforts are currently underway to migrate data to another publicly available site. **Equator Network Tool:** For original project evidence analysis: PRISMA Guideline. **AI Detailed Statement:** Machine learning models applied to Large Language Models (LLMs) were used in the article title/abstract screening process of the evidence synthesis step of this project as part of the SRDR+ platform. No other AI tools were used. The data that support the findings of this study were previously openly available in the US Agency for Healthcare Research and Quality Systematic Review Data Repository Plus (SRDR+) at https://doi.org/10.26300/572m-hh37 reference number 2847. Due to changes in US policy, SRDR+ was decommissioned in November 2025 (see Making Evidence Synthesis Data More Accessible and Reusable | Effective Health Care (EHC) Program). As of the time of writing, efforts are currently underway to migrate data from this platform to a different platform for public availability.

## SMART Learning Objectives

- Readers will gain an appreciation of the importance of formulating a system of logically related PECO questions to provide an abductive inferential basis for drawing conclusions regarding questions of complex environmental exposures.
- Readers will gain an understanding of the difference between an “inference to the best explanation” approach of evidence synthesis and a more typical “interventionist” inferential approach.

## Footnotes

1. We can imagine a scenario where military personnel are randomized not only to military occupational specialty (different occupational specialties have differential exposure likelihoods both within and outside a theater of conflict) but to deployment versus nondeployment (against current military policy). This would obviate the healthy warrior bias but would be unlikely to affect dilution bias in the exposed arm. Nor would this solve the challenge of the increase in tobacco use during conflict deployment. This is why, even with reference to the highly imaginative reference RCT, the effects of dilution bias (toward the null) and smoking (uncertain direction of bias) still must be methodologically handled in alternative ways (rating up the baseline in the former and used as the plausible alternative for the latter).
