# Supplementary material for: An abductive inferential system approach to weight of evidence syntheses: the problem and military exposures worked example
Source: Evid Based Toxicol. Author manuscript; Available in PMC 2026 Aug 8. (PMC13450199; doi:10.1080/2833373x.2025.2607260)
Supplement: Supplementary file 1 [file NIHMS2159704-supplement-Suppl_Material.docx]

Supplemental Material

An Inferential System Approach to Weight of Evidence Syntheses: The Problem and Military Exposures Worked Example

# The Importance of Plausible Alternatives

When a cause is purported to be an insufficient but necessary (i.e., there is at least one causal path where the exposure of interest is a necessary component even though the exposure cannot, of itself, explain the occurrence of the outcome) component of an unnecessary but sufficient cause (there are multiple causal paths, some of which do not include the exposure of interest), it is *logically necessary* to rule out the presence of another alternative cause that is both necessary and not entailed by the primary cause of interest.

To demonstrate this, we offer the following proof:

**Definitions**

- D = the candidate cause (deployment to SWATO)
- Q = the outcome (fibrotic lung disease)
- X = other factors required alongside D for sufficiency, e.g., genetic and adaptive immune (priming, reaction) conditions.
- A = alternative (disjunctive) sufficient conditions for Q (e.g., smoking, which could also be a condition set, (A∧X))
- Note: (A) may be conceived as any alternative exposure level explanation (cause) that would be necessary for Q. Thus, (A) may be conceived as (A_1, 2…n_).

**Premises**

1. (D∧X)∨A  ⟺  Q (Q occurs if and only if (D∧X) or (A) occurs)
2. Q→A (A is necessary for Q)
3. D alone and X alone are insufficient for Q
4. (A) may be absent from (D∧X)
5. D does not entail A (assumption for contradiction)

**Proof Steps**

**1. Assume (for contradiction): D does not entail A**

There exists at least one D such that A is false (D∧¬A). E.g., There is at least one instance of ILD occurring in deployed personnel where that person was never a cigarette smoker.

**2. Consider the sufficient condition (D∧X)**

By premise, (D∧X) is sufficient for Q.

Let’s instantiate X such that (D∧X) occurs, but still A is false (since A is not entailed by D, and may not be included in X). There exist formerly deployed non-smokers who were exposed to airborne hazards in theater and developed ILD.

**3. By sufficiency, Q must occur**

So (D∧X) is true ⇒ Q is true (from premise 1).

**4. By necessity, A must occur**

But Q→A, so if Q is true, A is true. If a deployed person developed ILD, they must of necessity have been a smoker.

**5. Contradiction**

Our scenario yields: A is false (from 1 and 2), yet A must be true (from 4).

This violates the law of non-contradiction: we cannot have both A and ¬A.

**6. Therefore, our assumption #2 is invalid.**

The only way to avoid contradiction is if whenever D (and X) occur, A also occurs—i.e., D

entails A. In other words, all deployers who develop fibrotic lung diseases must *also* have been smokers.

Thus, in order to demonstrate that deployment (combined with underlying conditions X) is an INUS condition for ILD (Q) we also have to rule out that having an ILD *requires* some other condition (A) that is necessary. Statistically, this would mean that, in all cases, after adjusting for (A) the relationship between (D∧X) and Q (deployment and ILD) does not approach zero.

Even if evaluating alternative explanations for INUS causes was not logically necessary, why would it still be important? First, and most importantly, we have a phenomenon that begs for an explanation: Why do we see greater levels of respiratory complaints among deployers than non-deployers (Doebbeling et al., 2000; Gray et al., 1999; Kang et al., 2000; Karlinsky et al., 2004; Kelsall et al., 2004; Saers et al., 2017; Skabelund et al., 2017)? To propose a single explanation only to determine that that explanation does not rise to the level of certainty leaves us in the in the awkward position of inconclusiveness or simply offering no explanation. Rather, as Lipton (Lipton, 2017) points out, we draw from a pool of *possible* explanations to identify the one that is the most credible in light of the evidence we have at hand. Secondly, operating from the best explanation provides a plausible basis for decision-making. Of course, this does not mean that the best explanation is guaranteed to be correct (new evidence may always surface to propose a different explanation), but it does provide us with a possible basis for intervening based on available evidence.

Within the military exposures research, while we could not find a plausible alternative explanation offered for increasing rates of ILD diagnoses among deployed military (Pugh et al., 2016) (with rates within the VA approximately 10 times higher than in the general population (Jeganathan & Sathananthan, 2024)), one possible alternative explanation could be the increase in smoking associated with deployment (Maccarone et al., 2024).

The challenge with using increased smoking during deployment as the primary alternative explanation for increased rates of respiratory conditions is that exposure to airborne hazards in the theater of operations and smoking are not mutually exclusive *competing* causes. Rather, both causes likely contribute to respiratory conditions and so our strategy was to determine whether, for each key question, the presence of smoking could *rule out* airborne hazard exposure as a plausible INUS explanation (an *insufficient* but *necessary* part of a condition which is itself *unnecessary* but *sufficient* for the result) (Shimonovich et al., 2021) (Figure S1). In other words, the goal was to determine whether deployment remained an important predictor of respiratory disorders among deployed personnel, even if smoking is assumed to be a contributing cause of some of the same respiratory disorders.

Figure S1. Alternative Evidence Scenario where Deployment is an INUS Condition of Respiratory Disorders

In Figure S1, each circle represents the sum total of multifactorial causes of respiratory disorders among deployed personnel (D=deployment, S=smoking, U=unmeasured causal factors). Because of the INUS conditions, either alternative condition could lead to respiratory disorders. However, from an evidence synthesis perspective, as long as the effect of exposure remains even after adjusting for smoking (and taking into account known biases toward the null), then exposure remains a plausible explanation.

Using this logic, we generated a series of key questions within each of the above inferential categories, specifying the warranted expectations if exposure to airborne hazards during deployment was a cause of respiratory disorders (H’) versus if increased smoking during deployment (H’’) could rule out the primary hypothesis. Following this approach, we formulated the key questions along with the corresponding expectations for each of the two explanations (Table S1).

Table S1. Key Questions Mapped to Inferential Criteria and Expectations Regarding Primary and Alternative Hypotheses

| **Inferential Category** | **Question** | **Direct Support if Consistent with the Following** | **Indirect Support if Inconsistent with the Following** |
| --- | --- | --- | --- |
|  |  | **H': Exposure to airborne hazards during deployment to SWATO increases the risk of the development of interstitial lung disease.** | **H'': Increases in smoking associated with deployment to SWATO fully explain the development of ILD among deployed personnel.** |
|  |  | Expected Pattern if H' is True | Expected Pattern if H'' is true. |
| **Antecedents** | What evidence exists to provide information on the constituents of airborne hazards in the Southwest Asia Theater of Operations (SWATO)? | PM levels will be higher in SWATO than would be expected in non-deployment environments. A range of airborne toxicants will be identified in the theater in locations with known troop deployment. | If higher levels of smoking explain the pattern of respiratory diseases seen in military personnel deployed to SWATO, we would not expect to see substantial differences in the pattern of toxicants present in SWATO versus the occupational environment of non-deployed soldiers. |
|  | What evidence exists that airborne hazard hazards present in the SWATO are toxic in humans? | Some of the specific airborne substances identified in SWATO will be identified as hazardous to human health by environmental agencies. |  |
| **Temporality** | Is there evidence of decreased respiratory health in military personnel following deployment to the SWATO compared to pre-deployment? | We would expect an increase in respiratory complaints following deployment compared to before deployment. Further out, we would expect an increase in longer-developing respiratory conditions. | We would expect an increase in respiratory complaints following deployment compared to before deployment. Further out, we would expect an increase in longer-developing respiratory conditions. |
| **Analogy/Co-Occurrence** | In the research on civilians, what is the evidence for an association between exposures to airborne hazards and interstitial lung diseases (ILDs)? | If the causal relationship proposed in H' is true, we would expect to see a pattern of relationship between purported cause and effect in other populations (civilian: occupational, population) | In samples balanced on smoking or in analyses that statistically adjust for smoking, we would expect that the effect of the primary exposure (pollution, occupational) of interest would be minimal to non-existent. |
| **Interaction** | Are known pathogens confirmed to be present in the lungs of SWATO deployers with the condition of interest? | We would expect the amount and constituents of PM in the lungs of deployers to be both higher and of different composition than typical non-deployed individuals (civilians or non-deployed military). We would expect these differences to map onto differences in outcome status. | We would expect to see differences in PM infiltrates between deployers and non-deployers, but these differences should not be associated with the outcome of interest. Rather, we would expect there to only be differences in the outcomes based on smoking status. |
| **Mechanism** | In in vitro and in vivo research, what is the evidence for mechanistic linkages between military-relevant exposure profiles of personnel deployed to the SWATO and inflammatory and fibrotic processes in the lungs? | In vivo and in vitro research will identify specific biological pathways between exposure to SWATO-relevant PM and inflammatory and fibrotic processes. These will include for example: pro-inflammatory pathways, mitochondrial dysfunction, AhR pathways (due to exposure to PAHs), cytochrome P450 enzyme induction, and epigenetic changes that differ from smoking-related changes. | In vivo and in vitro research will identify specific biological pathways and processes between smoking and inflammatory and fibrotic processes that differ from pathways related to fine PM or PAH, VOCs, etc.: e.g., Nicotinate and nicotinamide pathway, macrophage polarization, autophagy suppression, mTOR pathway, and epigenetic changes that differ from fine PM-related (and PAH, VOC-related) changes. |
|  | In human biomarker research, what is the evidence that airborne exposures during military deployment to the SWATO result in measurable biomarker differences between deployed versus non-deployed military? | For exposure at one point in time to have a longer-term effect, we would expect to find biomarker changes (e.g., biological markers of transcriptional changes) both from pre- to post-deployment as well as differences between deployers and non-deployers. | We would expect biomarker changes to be those associated with smoking. We would expect biomarker changes to be similar between deployed and non-deployed smokers and deployed and non-deployed non-smokers. |
|  | In human biomarker research, what is the evidence that airborne exposures during military deployment to the SWATO result in changes in biomarkers implicated in pro-inflammatory and pro-fibrotic pathways (e.g., change in expression of cytokines or miRNAs associated with disease risk)? | We would expect some of the changes in biomarkers to be associated with increases in ILD risk (specifically via pathways linked to inflammation and fibrosis). | We would expect changes associated with biomarkers related to smoking to be associated with pathways linked to inflammation and fibrosis but not expect non-smoking-related pathways to be implicated. |
| **Specific Differences** |  |  |  |
| **Differences in symptoms** | Are military deployed to the SWATO more likely to complain of respiratory problems following deployment compared to those who did not deploy to the SWATO? | Patients experience symptoms (rather than diseases or diagnoses). Thus, we would expect a higher level of complaints regarding respiratory symptoms in deployed versus non-deployed military personnel. | We would expect any differences in respiratory complaints between deployers and non-deployers to disappear after adjusting for smoking. |
| **Differences in lung pathology** | Are the lungs of military post-deployers to the SWATO seeking treatment post-deployment for respiratory complaints histopathologically distinct from: | We would expect a range of histopathological differences between deployers and various other non-deployed groups. |  |
|  | Healthy individuals | We would expect a greater proportion of histopathological features in deployers compared to healthy individuals assuming smoking status is balanced or adjusted for. | We would expect histopathological differences to be associated with smoking status and not deployment status. In imbalanced samples where smoking is more prevalent in healthy individuals, we would expect a higher level of histopathological features in the arm with the greatest proportion of smokers. |
|  | Military personnel who did not deploy to the SWATO | We would expect a greater proportion of histopathological features in deployers compared to non-deployers assuming smoking status is balanced or adjusted for. | We would expect histopathological differences to be associated with smoking status and not deployment status. In imbalanced samples where smoking is more prevalent in healthy individuals, we would expect a higher level of histopathological features in the arm with the greatest proportion of smokers. |
|  | Individuals with other respiratory diseases | We would expect different histopathological patterns in deployed individuals compared to non-deployers with other respiratory diseases. | We would expect different histopathological patterns in smokers compared to non-smokers with other respiratory diseases. |
| **Differences in diagnoses** | Are military personnel deployed to the SWATO more likely to receive an ILD diagnosis following deployment than those who did not deploy to the SWATO? | We would expect differences in the patterns of diagnoses of interstitial lung diseases (ILDs) among deployers and non-deployers. However, because ILDs are rare events, we would be surprised to find statistically significant differences except in very large samples. Additionally, because even non-deployed military personnel are exposed to airborne hazards in the line of service (e.g., jet fuels, high PM, mold, solvents and pesticides, higher levels of smoking) we would not necessarily expect the absolute rate to be higher for all ILD diagnoses, though we would expect the pattern of diagnoses to be different for deployed versus non-deployed military. | We would expect patterns of ILD diagnoses to be associated with smoking status and not deployment status. |

Abbreviations: PM: particulate matter; SWATO: Southwest Asia theater of operations; ILD: interstitial lung disease; AhR: Aryl hydrocarbon Receptor pathways; PAH: polycyclic aromatic hydrocarbon; VOC: volatile organic compound; mTOR pathway: mammalian target of rapamycin pathway

Doebbeling, B. N., Clarke, W. R., Watson, D., Torner, J. C., Woolson, R. F., Voelker, M. D., Barrett, D. H., & Schwartz, D. A. (2000). Is there a Persian Gulf War syndrome? Evidence from a large population-based survey of veterans and nondeployed controls. *Am J Med*, *108*(9), 695–704. <https://doi.org/10.1016/s0002-9343(00)00405-8>

Gray, G. C., Kaiser, K. S., Hawksworth, A. W., Hall, F. W., & Barrett-Connor, E. (1999). Increased postwar symptoms and psychological morbidity among US Navy Gulf War veterans. *The American journal of tropical medicine and hygiene*, *60*(5), 758–766.

Jeganathan, N., & Sathananthan, M. (2024). Interstitial Lung Disease Cases, Prevalence Rates and Trends Among States in the United States. *Chest*, *165*(2), 389–395. <https://doi.org/10.1016/j.chest.2023.08.006>

Kang, H. K., Mahan, C. M., Lee, K. Y., Magee, C. A., & Murphy, F. M. (2000). Illnesses among United States veterans of the Gulf War: a population-based survey of 30,000 veterans. *J Occup Environ Med*, *42*(5), 491–501. <https://doi.org/10.1097/00043764-200005000-00006>

Karlinsky, J. B., Blanchard, M., Alpern, R., Eisen, S. A., Kang, H., Murphy, F. M., & Reda, D. J. (2004). Late prevalence of respiratory symptoms and pulmonary function abnormalities in Gulf War I veterans. *Archives of internal medicine*, *164*(22), 2488–2491.

Kelsall, H. L., Sim, M. R., Forbes, A. B., McKenzie, D. P., Glass, D. C., Ikin, J. F., Ittak, P., & Abramson, M. J. (2004). Respiratory health status of Australian veterans of the 1991 Gulf War and the effects of exposure to oil fire smoke and dust storms. *Thorax*, *59*(10), 897–903.

Krieger, N. (2008). Proximal, distal, and the politics of causation: what's level got to do with it? *Am J Public Health*, *98*(2), 221–230. <https://doi.org/10.2105/AJPH.2007.111278>

Lipton, P. (2017). Inference to the best explanation. *A Companion to the Philosophy of Science*, 184–193.

Maccarone, J. R., Sterns, O. R., Timmons, A., Korpak, A. M., Smith, N. L., Nakayama, K. S., Baird, C. P., Ciminera, P., Kheradmand, F., Fan, V. S., Hart, J. E., Koutrakis, P., Jerrett, M., Kuschner, W. G., Ioachimescu, O. C., Montgrain, P. R., Proctor, S. P., Redlich, C. A., Wendt, C. H.,…Wan, E. S. (2024). Deployment-related Cigarette Smoking Behaviors and Pulmonary Function Among U.S. Veterans. *Mil Med*. <https://doi.org/10.1093/milmed/usae049>

Pugh, M. J., Jaramillo, C. A., Leung, K.-w., Faverio, P., Fleming, N., Mortensen, E., Amuan, M. E., Wang, C.-P., Eapen, B., & Restrepo, M. (2016). Increasing prevalence of chronic lung disease in veterans of the wars in Iraq and Afghanistan. *Military Medicine*, *181*(5), 476–481.

Reiss, J. (2012). Causation in the sciences: An inferentialist account. *Studies in history and philosophy of science. Part C, Studies in history and philosophy of biological and biomedical sciences*, *43*(4), 769–777. <https://doi.org/10.1016/j.shpsc.2012.05.005>

Saers, J., Ekerljung, L., Forsberg, B., & Janson, C. (2017). Respiratory symptoms among Swedish soldiers after military service abroad: association with time spent in a desert environment. *European Clinical Respiratory Journal*, *4*(1), 1327761.

Schünemann, H., Hill, S., Guyatt, G., Akl, E. A., & Ahmed, F. (2011). The GRADE approach and Bradford Hill's criteria for causation. *J Epidemiol Community Health*, *65*(5), 392–395. <https://doi.org/10.1136/jech.2010.119933>

Schunemann, H. J., Tugwell, P., Reeves, B. C., Akl, E. A., Santesso, N., Spencer, F. A., Shea, B., Wells, G., & Helfand, M. (2013). Non-randomized studies as a source of complementary, sequential or replacement evidence for randomized controlled trials in systematic reviews on the effects of interventions. *Res Synth Methods*, *4*(1), 49–62. <https://doi.org/10.1002/jrsm.1078>

Shimonovich, M., Pearce, A., Thomson, H., Keyes, K., & Katikireddi, S. V. (2021). Assessing causality in epidemiology: revisiting Bradford Hill to incorporate developments in causal thinking. *Eur J Epidemiol*, *36*(9), 873–887. <https://doi.org/10.1007/s10654-020-00703-7>

Skabelund, A. J., Rawlins, F. A., 3rd, McCann, E. T., Lospinoso, J. A., Burroughs, L., Gallup, R. A., & Morris, M. J. (2017). Pulmonary Function and Respiratory Health of Military Personnel Before Southwest Asia Deployment. *Respir Care*, *62*(9), 1148–1155. <https://doi.org/10.4187/respcare.05438>

Whaley, P., Piggott, T., Morgan, R. L., Hoffmann, S., Tsaioun, K., Schwingshackl, L., Ansari, M. T., Thayer, K. A., & Schunemann, H. J. (2022). Biological plausibility in environmental health systematic reviews: a GRADE concept paper. *Environ Int*, *162*, 107109. <https://doi.org/10.1016/j.envint.2022.107109>
